# Conducting Psychosocial Intervention Research among Adolescents and Young Adults with Cancer: Lessons from the PRISM Randomized Clinical Trial

**DOI:** 10.3390/children6110117

**Published:** 2019-10-24

**Authors:** Abby R. Rosenberg, Courtney C. Junkins, Nicole Sherr, Samantha Scott, Victoria Klein, Krysta S. Barton, Joyce P. Yi-Frazier

**Affiliations:** 1 Palliative Care and Resilience Lab, Seattle Children’s Research Institute, Seattle, WA 98101, USA; courtney.junkins@seattlechildrens.org (C.C.J.); Nicole.Sherr@seattlechildrens.org (N.S.); Samantha.Scott@du.edu (S.S.); Krysta.Barton@seattlechildrens.org (K.S.B.); joyce.yi-frazier@seattlechildrens.org (J.P.Y.-F.); 2 Department of Pediatrics, University of Washington School of Medicine, Seattle, WA 98105, USA; 3 Department of Psychosocial Oncology and Palliative Care, Dana Farber Cancer Institute, Boston, MA 02101, USA; vklein013@gmail.com; 4 Department of Pediatrics, Boston Children’s Hospital, Boston, MA 02101, USA

**Keywords:** psychosocial, clinical trial, adolescent and young adult, cancer, palliative care

## Abstract

Background: Adolescents and young adults (AYAs) with cancer have poor psychosocial outcomes, in part because their limited participation in clinical trials precludes intervention-testing. We previously reported results of a successful randomized trial testing an AYA-targeted psychosocial intervention. Here, we aimed to describe strategies learned during the trial’s conduct. Methods: We summarized data from the medical record and staff field notes regarding reasons for participation/non-participation. We conducted two focus groups with study staff; directed content analyses identified strategies for success. Results: 92 AYAs enrolled (77% of approached; *n* = 50 Usual Care (control), *n* = 49 PRISM (intervention)). In eligible families who declined participation (*n* = 22 AYAs, *n* = 8 parents), the AYAs more commonly had advanced cancer (*n* = 11 (37%) declined vs. *n* = 25 (26%) enrolled). AYA reasons for non-enrollment were predominantly “not interested”; parents worried participation was “too burdensome.” Staff strategies for accrual included having significant time to introduce the study and underscoring a desire to learn from the patient. After enrollment, AYAs who discontinued participation were more commonly assigned to control (*n* = 5 (10%) control vs. *n* = 2 (4%) intervention). Only *n* = 1 AYA chose to discontinue participation after receiving the intervention. Conclusions: Efforts to engage AYAs prior to and during studies may help with accrual and retention.

## 1. Introduction

Adolescents and Young Adults (AYAs) with cancer have disproportionately poor physical and psychosocial outcomes compared to younger pediatric and older adult counterparts [1,2]. Reasons for these poor outcomes include differences in cancer biology, the contribution of developmental life-challenges to the burden of cancer, and a lack of AYA participation in clinical trials [3]. Indeed, the latter is key; without rigorous testing of AYA-targeted interventions, we cannot improve AYA outcomes [4,5,6].

While most AYAs believe participation will help themselves or others, fewer than 50% enroll in a trial during their cancer experience [4,7,8,9]. Barriers may be concrete, including sociodemographic factors and limited access to trials, as well as philosophical, including AYA trust in research processes, practical considerations for participation, and perceptions of trial acceptability [9,10,11,12,13]. These barriers are particularly important when it comes to psychosocial intervention research, where even fewer AYAs participate compared to pharmacology-based research [11]. AYAs cite pragmatic considerations of time commitments and the stigma associated with mental health services [1,14,15,16,17,18,19]. Indeed, even clinical (non-research based) psychology services are rarely utilized by AYAs; the average refusal rate for traditional cognitive behavioral therapy in adolescent chronic disease settings is 37%; subsequent attrition is up to 32% [20]. Finally, timing is also a challenge. Despite evidence that cancer-related distress occurs early and often during cancer therapy, most interventions begin when that therapy is complete (e.g., during AYA “survivorship”) [21,22,23,24,25]. 

We recently conducted and reported the results of a successful randomized clinical trial (RCT) testing the impact of a novel psychosocial intervention among AYAs who had recently received a diagnosis of new or recurrent cancer [26,27,28]. Briefly, we enrolled and randomly assigned *n* = 92 AYAs with new or recurrent cancer to receive usual psychosocial care with or without the Promoting Resilience in Stress Management (PRISM) intervention. We found that AYAs who received PRISM reported higher resilience, hope, benefit finding, and cancer-related quality of life, and lower psychological distress 6 months after enrollment [26,27,28]. The purpose of the present report is to provide detailed descriptions of our study procedures and the lessons we learned during the conduct of this trial. Our goal is to share our challenges and learned strategies, in turn informing other AYA oncology investigators and, we hope, supporting AYA clinical trial participation more broadly. 

## 2. Materials and Methods

Design & Setting: This phase 2, parallel, randomized clinical trial (RCT) was conducted at Seattle Children’s Hospital (SCH) between January 2015 and October 2016. The SCH Institutional Review Board approved the study, including its procedures for screening and enrollment. The primary objective was to determine if a novel psychosocial intervention (“Promoting Resilience in Stress Management,” PRISM) was associated with improved AYA patient-reported resilience 6-months following enrollment (clinicaltrials.gov NCT02340884). Secondary objectives included examining PRISM’s effect on AYA patient-reported quality of life, hope, benefit finding, distress, anxiety, and depression. Results have been reported elsewhere; briefly, PRISM was associated with improvements in all outcomes of interest [26,27,28]. The present report describes our team’s experience and lessons learned during the conduct of this RCT. 

RCT Participants: AYA patients were eligible if they were: (i) 12–25 years old; (ii) fluent in English; and, (iii) either diagnosed with a new cancer between 1 and 10 weeks prior to enrollment, OR, ever diagnosed with “advanced cancer” (defined as progressive, recurrent, or refractory disease). AYAs with cognitive disabilities precluding written or verbal communication were excluded. 

The PRISM Intervention: PRISM’s development has been described elsewhere, including its theoretical underpinnings, feasibility, and acceptability [29,30]. Briefly, it is grounded in stress-and-coping and resilience theories, and was designed through iterative patient-centered processes [30]. It is deliberately brief, skills-focused, and deliverable by trained, bachelors-level non-clinical staff [29]. 

PRISM’s content includes four required and one optional, 30–50 min, 1-on-1 sessions delivered approximately every other week [29]. Session 1 (“Stress management”) focuses on relaxation and mindfulness skills such as deep breathing and meditation techniques. Session 2 (“Goal-setting”) teaches AYAs to create “SMART” (Specific, Measurable, Actionable, Realistic, and Time-dependent) goals. Session 3 (“Positive Reframing”) helps participants recognize negative self-talk and corresponding emotions and reframe them realistically and/or positively. Session 4 (“Benefit finding”) teaches AYAs how to find meaning or gratitude from difficult situations, including cancer. Session 5 (“Coming Together”) is optional and involves a facilitated family meeting where participants may share learned skills with loved ones. Between sessions, participants receive worksheets to practice skills. The same person delivers all sessions to a given AYA. Each session is administered in a private patient-room, during inpatient hospital stays or before/after outpatient clinic visits. 

Study Staff: During the 22 months of this trial, seven women clinical research coordinators (CRCs) each received 8 h of standardized training in PRISM’s delivery, including directed roleplay scenarios and fidelity-monitoring. All staff were college-graduates. One was also a candidate for a PhD in Psychology, one held a Master of Public Health degree, and one was an Advanced Registered Nurse Practitioner (ARNP). The other four had no graduate-level training. At any given time, only one woman worked on the project full-time; the effort of other CRCs ranged from 5–10%, for a total of 100–125% staff full-time equivalent (FTE) during the course of the study. All CRC funding for this study was provided by the two grants supporting the project.

Screening, Approach, and Enrollment: In our “Palliative Care and Resilience” Lab at Seattle Children’s Research Institute, we developed standard procedures for screening, approach, and enrollment based on 5 years of experience in pediatric psychosocial research. These standards were in place at the time the present study launched. Briefly, all CRCs are trained to screen and approach patients/families for all open studies. This sharing of responsibilities enables at least one CRC to be present at the hospital and adjacent clinics all day, Monday-Friday. The lead CRC reviews clinic rosters and inpatient registries at least once weekly to identify eligible patients. During a weekly team meeting, she and other CRCs determine who will approach each patient/family and when. For the present study, this determination included: (i) review of proximal clinical appointments and procedures; and, (ii) review of medical and psychosocial clinical notes. The purpose of these chart reviews was to consider patient and family care-burdens; for example, if an AYA had a busy schedule with physical therapy plus an immediate social crisis at home, the CRCs might defer approaches. Alternatively, if the AYA had a lab appointment and then a wait-time of an hour before seeing a clinician, CRCs would plan to approach during that hour. In all cases, CRCs conferred with clinical staff (Medical Assistants, physicians, or bedside nurses) to confirm timing of approach. 

CRCs approached AYAs in person. When the AYA was under 18 years old, they approached only if a parent/guardian was present. All CRCs were trained to introduce the study with standardized phrases, beginning with a request for permission to talk about a potential research study, and followed by a brief rationale of the project: *“Over the past few years, we worked with other teens and young adults with cancer to learn what helped them cope with it. Then, we turned what we heard into a program we can teach to other patients. Now, we are trying to figure out if that program works, and we really want to learn from people like you. Can I tell you more about the project?”*

If AYAs were willing to hear more, the CRC continued to explain the nature of the research study, including the concept of randomization with a “coin toss” metaphor, and the study requirements for each randomization arm. CRCs included a brief statement about incentives: *“Regardless of the coin toss, as a thank you for your time, we will give you a $25 gift card after the first survey, and a $50 card after the 6- and 12-month surveys.”*

Whenever possible, CRCs assessed AYA interest first, and parent interest second. If AYAs were under 18 years old and either party said “no,” the AYA was not enrolled. When there was a disagreement, however, CRCs asked to hear each stakeholder’s reasons for/against the study and recorded the reason(s) in their field notes. They then asked if the family wanted to discuss it and if so, they (the CRCs) could come back.

In all cases, CRCs asked the AYA and family if they would like to review the consent and think about the project. If the AYA/family said “yes,” the same CRC returned to the bedside up to twice more to continue discussion. Continued request to review or think after three visits was considered a “passive” refusal. CRCs then thanked families for their consideration and stated they would not return again. 

When AYAs and parents/guardians verbally agreed, the CRC conducted a formal consent conference. Using the written consent as a guide, she reviewed the study procedures, risk and benefits of participation, and limits of confidentiality. Specifically, she shared that any concerns regarding AYA self-harm or harm to others would be reported to the parents and/or clinical staff. All AYAs under 18 years old provided written informed assent and their parents or legal guardians provided written informed consent. AYAs 18 years old and older provided written consent. When an AYA turned 18 during the course of the study, he/she was re-approached to confirm continued interest and obtain written consent. 

Following written assent/consent, AYAs were immediately registered within a secure database and randomized 1:1 to Usual Care (UC) or UC plus PRISM. The CRC returned to the bedside to: (i) deliver copies of the consent form(s); (ii) deliver baseline surveys; and (iii) inform the family of the AYA’s assignment. If the AYA was assigned to PRISM, the CRC also queried availability and preferences for PRISM sessions. For example, she and the family reviewed the AYA’s clinical schedule to coordinate the first PRISM session with a planned outpatient clinic visit or inpatient hospital stay. Following this discussion, the CRC team identified who was available for PRISM delivery. Considerations included current PRISM “case-loads,” the AYA/family scheduling preferences (e.g., day of the week the CRCs were available), and other staff responsibilities. The CRC who conducted the approach and consent conference was not necessarily the same who delivered the intervention. 

Study Procedures: Surveys consisted of age-validated patient-reported outcome measures (131 total items) and were requested upon enrollment, at 6-months (primary outcome), and 12-months (exploratory outcome) [26,27]. In addition, a brief (16-item) survey was requested at months 2 and 4. Medical and sociodemographic characteristics were extracted from the medical record.

Baseline surveys were invited in person immediately following randomization; in all cases, the CRC provided a paper-pencil option, and offered a digital copy via email (REDCap) as an alternative. Few AYAs completed surveys immediately; staff contacted participants once weekly for up to 3 weeks to collect them. Surveys not completed within that timeframe were considered missing. Follow-up surveys (both brief and long) were also offered as paper-pencil or digital, however, as participants were not consistently in clinic or the hospital when these were due, CRCs contacted participants in person or by phone to determine their preference. Participants received reminders 7 days prior to each follow-up survey-due date and again once weekly for up to three weeks following their due dates. 

Mixed Analytic Methods for the Present Analysis: We used quantitative data from CRC field notes and the medical record to summarize characteristics of participants who did and did not enroll, and those who enrolled and did/did not complete surveys. We also calculated the timing and response rate of approach procedures. 

Other analyses were qualitative and followed standards for reporting qualitative research (SPQR) [31]. First, two senior investigators with formal training in health services and qualitative methods (ARR and KSB) used directed content analyses to code CRC field notes, including salient reasons for non-participation [32]. For context, one of the coders (ARR) is also the PI of the study and an AYA oncologist who may have interacted with patients and families enrolled in this study; the other coder (KSB) has extensive experience with AYA psychosocial research conduct and qualitative analysis, and had no contact with patients enrolled in this study. 

Following primary coding, both investigators (ARR and KSB) co-conducted two focus groups with a convenience sample of study CRCs; four of the seven CRCs who worked on this study were available for focus group participation. Each focus group was conducted in-person, with video-conference options for CRCs who were not on-site. In the first focus group, quantitative summary statistics and preliminary themes from field notes were presented to CRCs for feedback and triangulation of findings [33]. In the second, a semi-structured interview guide included queries regarding learned strategies for screening, approaching, and enrolling patients, impressions of barriers to data collection, and what they would recommend to new staff. Following the second focus group, ARR and KSB compared new field notes, developed new codes, and conducted new directed content analyses of focus group data [32]. Findings, in the form of summarized “lessons learned,” were reported back to CRCs in writing for triangulation and consensus [33]. Finally, all findings, including tables, figures, and the draft manuscript were shared with two study CRCs (NS and VK), a CRC who did not work on the present study (SS), and our senior PRISM trainer (CJ) to confirm accuracy, trustworthiness, and applicability [31]. 

## 3. Results

Screening, Approach, and Enrollment: After screening 483 potential participants, 353 were determined to be ineligible [26,27]. The predominant reasons for ineligibility included the fact that AYAs were receiving primary cancer treatment elsewhere (*n* = 156 of 353 ineligible AYAs, 44%) or that they had a diagnosis other than cancer (*n* = 117, 33%). In 17 cases (5%), we were unable to introduce the study and obtain consent because a legal parent or guardian was not at the bedside. Eight (2% of) eligible AYAs died before we were able to introduce the study.

**Figure 1:**
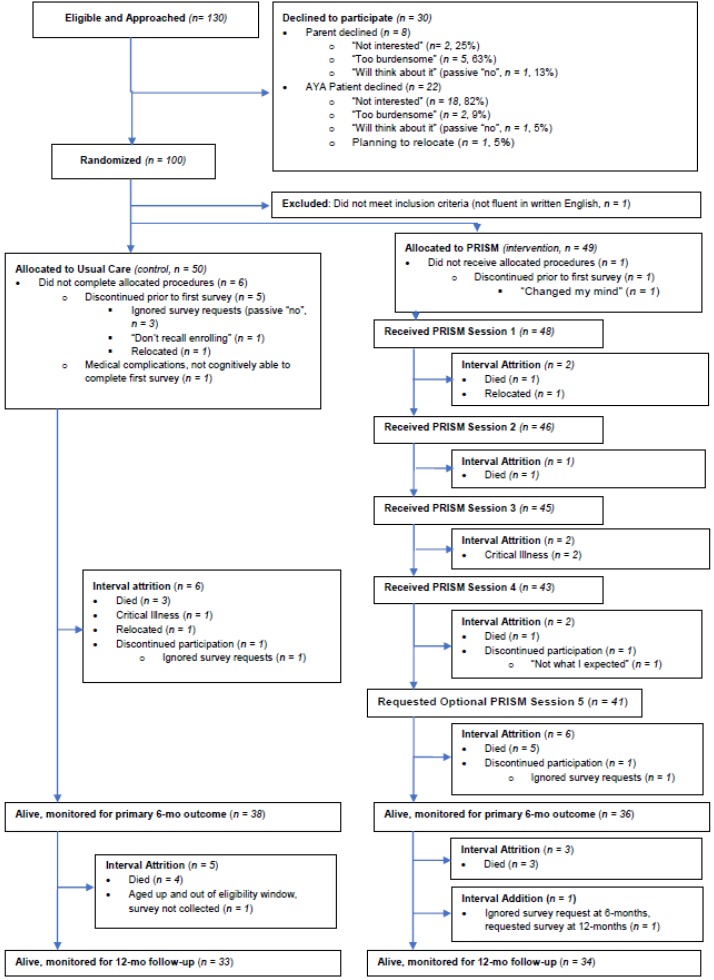
Flow of screening, approach, enrollment, and attrition during the course of the Promoting Resilience in Stress Management (PRISM) randomized trial.

We approached 130 eligible participants to describe the study and query their interest in participation (Figure 1). Of these, 30 (23%) declined participation, including 8 parents whose AYAs were median aged 13.5 years (range 12–16). The most common reason parents declined participation (*n* = 5, 65%) was a sense that participation was “too burdensome” for their AYA-aged child. This explanation was given for both younger AYAs (*n* = 2 who were 12 years old) and older AYAs (*n* = 3 who were 15–16 years old). 

Twenty-two AYAs (median aged 15.5 years, range 12–24) declined participation (Figure 1). Their most common reason (*n* = 18, 82%) was disinterest. Upon deeper probing, *n* = 7 (32%) shared they did not “need” or “believe in” psychosocial supports such as those the intervention would offer, if they were randomized to the intervention arm (Table 1). Only 2 (9%) felt that the program itself would be burdensome, either due to the length of surveys or the time involved with the intervention. In several cases, parents encouraged the AYA to participate and/or expressed regret that the AYA had declined, stating their own opinion that such programs were helpful, if not necessary. All parents respected their AYA’s ultimate decision, however, agreeing that the AYA’s engagement and interest were paramount.

Enrollment rates were at least 75% for most of the study; they ranged from 75% to 94% between January 2015 and July 2016 and were 58% during the last 3 months of the study (August 2016–October 2016). AYAs who chose not to enroll were generally similar to those who did, with some exceptions (Table 2). Participants who enrolled and did not complete baseline surveys were more commonly male and/or of non-White race. AYAs with advanced cancer less commonly enrolled and, when enrolled, less commonly completed the baseline survey.

The optimal timing of approach was also different for AYAs with new or advanced cancer. Among those with diagnoses of new malignancies, enrollment rates were 79% and 80% within weeks 1–5 and weeks 6–10 after diagnosis, respectively (Table 3). In contrast, for those diagnosed with recurrent, refractory, or progressive disease, enrollment rates were 56% 1–5 weeks after hearing the news, compared to 85% 6–10 weeks after the news. 

Attrition Between Randomization and Baseline Data Collection: One hundred AYAs were randomly assigned to PRISM (intervention, *n* = 50) or Usual Care (UC, control, *n* = 50, Figure 1 and Table 2). We learned after his randomization that one PRISM-participant was ineligible due to lack of fluency in written English; he was subsequently removed from the study. Of the remaining 49 AYAs assigned to PRISM, *n* = 1 (2%) did not complete the baseline survey, stating he “changed his mind” once he saw the length of the survey. Of the *n* = 50 UC AYAs, most shared they were disappointed not to receive PRISM. Six (12%) did not complete the baseline survey, including *n* = 4 (8%) by choice: three did not respond to our requests and were labeled as “passive” refusals, and 1 stated she did not remember enrolling and was no longer interested.

Uncontrollable Attrition Due to Death and Critical Illness: Among the 99 enrolled and randomized participants, *n* = 15 (15%) died or experienced serious medical complications precluding their ability to complete the primary outcome survey at 6-months. These included *n* = 10 (20%) of the AYAs assigned to PRISM and 5 (10%) of the AYAs assigned to UC. By the 12-month follow-up point, an additional 7 AYAs had died (*n* = 3 and *n* = 4, respectively), for a total attrition due to critical illness or death of n = 22 (22%) in one year. 

Participant-Driven Attrition among Intervention Recipients: Of the *n* = 48 AYAs assigned to PRISM and with baseline survey data, only one chose to discontinue participation once the program started (Figure 1). This patient received all 4 main sessions and then requested to discontinue his participation, stating the program was “not what I expected.” Although the fifth session was optional, all other AYAs chose to receive it. Among the 37 AYAs who were alive and able at the 6-month follow-up, *n* = 1 (3%) passively refused to complete the 6-month study survey and all others (*n* = 36, 97%) completed it. At 12-months, the same AYA who had passively refused her 6-month survey asked to complete a new one. She and all other able AYAs completed the final survey.

Participant-Driven Attrition among Control Recipients: Among the 38 who were alive and able at the 6-month follow-up, *n* = 1 (3%) passively refused to complete the survey and all others (*n* = 38, 97%) completed it. Between the 6- and 12-month time-points, one AYA turned 18 and we were not able to reach him to obtain renewed informed consent. All other able AYAs completed the final survey.

*Participant Preferences for Survey Modality: At* Baseline, *n* = 69 (75%) of participants chose to complete surveys via paper-pencil versus digitally via email. Over time, however, these preferences shifted. At 6- and 12-month follow-up survey-requests, *n* = 24 (32%) and *n* = 9 (13%) requested paper-pencil, respectively. Focus group discussions with CRCs suggested these findings were driven by convenience for both AYAs and study-staff. For example, CRCs handed participants paper-pencil options immediately following enrollment, when AYAs were in clinic or hospital rooms. Not only was it more convenient for staff to quickly return to collect the paper-surveys, it was also easier for patient participants to complete them in sections while waiting for clinical providers or procedures. In contrast, at follow-up time-points, fewer AYAs were in clinic or the hospital and email may have been easier. 

Lessons Learned: CRC Focus Group discussions centered on what staff learned during the course of the study, and what they would recommend to future investigative teams (Table 4). We were unable to detect differences in CRC-reported lessons learned based on their FTE, discipline, or other roles within our team. With respect to the approach process, CRCs universally endorsed the needs for flexibility and patience to work around patient and family schedules. They also underscored collaboration with clinical staff and the necessity of developing a fundamental knowledge of medical vocabulary so that they could appreciate patient clinical experiences. 

CRCs also shared that they learned how to explore AYA or family hesitation, and that this exploration often translated to study participation (Table 4). One CRC said, “*we learned that specific reasons for hesitation were often addressable.*” For example, when AYAs or families said they were not interested, staff would say, “*thank you for telling me that. Can I ask why?*” Likewise, if an AYA or parent said something about the burden of participation, the CRC explained that the intervention was delivered in tandem with scheduled clinic visits or hospital stays, so “*there are no extra appointments for you*.” Many CRCs elaborated by saying the sessions would be delivered during “down time,” when the AYA was waiting for a blood draw in the clinic or otherwise “stuck in the hospital.” If the AYA cited stigma about mental health interventions, the CRC clarified that PRISM is “*skills not therapy*.” I the AYA said something about already coping well and not needing the intervention, CRCs replied, “*that is great! We want to learn from people who struggle AND those who don’t. That means you are still eligible and we would love to learn from you.*” 

A related observation was that invoking the voice of the patient encouraged enrollment. “*Saying ‘we really want to learn from you’ was really powerful,*” said one CRC. Few CRCs felt that gift-card incentives drove enrollment, although most highlighted them when an AYA seemed disengaged. “*When the teen’s eyes glazed over, sometimes I would bring up the incentive earlier to see if it would get him back into the conversation*.” 

Most CRCs commented that it was challenging to navigate the medical system without basic medical knowledge. They learned through trial and error how to read a chart. When the PI began meeting with staff weekly, for the sole purpose of describing patients’ medical cases and contexts, staff felt more supported. 

Last, the team’s diverse roles and levels of study-commitment were both a help and a hindrance. One CRC who worked part time felt less engaged in the work. When she became more involved in regulatory and other study activities, she also felt more committed. The ARNP experienced conflicting demands in her clinical and research responsibilities. Another CRC stated that sharing approaches and intervention delivery diversified her responsibilities, translating to greater job satisfaction. All team members stated that the multidisciplinary team made both the study and the global team stronger.

## 4. Discussion

AYA participation in clinical trials is limited, especially when the trial involves a psychosocial intervention [11]. We aimed to share our experiences and lessons learned in the successful conduct of the PRISM randomized trial [26,27,28], We found that investing in staff and engaging AYAs were key. Staff investment included full-time personnel devoted to the project, extensive training in pre-enrollment procedures such as describing the study, and ongoing support to help staff navigate the medical system. AYA engagement included establishing rapport early, exploring patient-hesitations about study participation, emphasizing a wish to learn from AYAs directly, and, for the intervention arm, delivering a patient-centered program. Indeed, PRISM was designed by AYAs for AYAs [29]. Whereas AYAs assigned to control were more likely to voluntarily discontinue participation, only 1 AYA who started the intervention did the same. 

In many cases, staff investment and AYA engagement strategies were synergistic. For example, CRCs reported spending significant time waiting for AYAs to be “ready” for an approach, and this ability to meet patients on patients’ terms translated to higher enrollment rates. Likewise, training CRCs with guides to introduce the study and explore a family’s hesitation both enabled CRC confidence and rapport-building.

Adolescence and Young Adulthood is characterized by emerging autonomy and identity development. It is therefore not surprising that direct patient-engagement facilitated participation. Like others, we found that including AYAs in consent discussions, exploring their concerns about study requirements, addressing perceived stigmata of psychosocial interventions, and emphasizing how their contributions may help others all promoted enrollment [7,8,9,13]. Our approach may have been additionally powerful because the study was designed to be as convenient as possible for AYAs, including flexibility in intervention delivery and format of surveys.

Our design was not powerful enough, however, to overcome other known barriers to clinical research participation [10,11,23,24,34]. AYAs with more advanced cancer were less likely to enroll or complete surveys, despite the fact that they represent a highly vulnerable population in need of psychosocial support [35,36,37,38,39,40,41,42,43,44,45]. Although there were more young men than women in our study, young men were still less likely to enroll or complete surveys. While non-White AYAs more commonly enrolled, they were less likely to complete surveys. This pattern may be due to social desirability biases, differences in health literacy and cultural views about research, and additional burdens of survey completion for more vulnerable populations. Efforts must be made to understand and overcome these barriers. 

The limitations of our findings suggest additional barriers. First, this trial was conducted at a single, large AYA center. We had a sufficiently large patient population to justify a full-time CRC for this project. Second, the number of trials at our center translated to a large team of CRCs who could support one another and share the burdens of approach. These limitations are important for smaller centers, where there may be fewer (or part-time) CRC staff, and where it is less feasible for staff to remain in clinical spaces and wait for convenient times to approach AYAs. Smaller centers may need to consider cross-training research staff across multiple types of studies, partnering with clinical staff for study outreach and discussion, and/or identifying a “research champion” responsible for facilitating clinical trial information dissemination and AYA participation. Third, because PRISM was founded at our center, clinical and research staff were highly familiar and supportive of the program. Other sites might find psychosocial intervention delivery more challenging due to higher patient/family stigma and/or limited staff support. Taken together, our findings may not be reproducible.

## 5. Conclusions

We aimed to describe our approach, experience, and lessons learned during the PRISM randomized trial. We found that support for both staff and AYA patients mattered. In particular, having full-time staff dedicated to the study and investing in staff training in pre-study procedures like recruitment promoted enrollment. Likewise, enabling patient engagement by exploring AYA concerns, underscoring their value as research participants, and making the study convenient were all important. While additional barriers to AYA participation in clinical research remain, we hope these lessons learned will help future teams, and our larger AYA oncology community continue to learn and develop programs for an important population of young people. 

## Figures and Tables

**Table 1:** Adolescents and young adults (AYAs) and Parent Preferences for Research Participation.

Population	Sample Explanations and Preferences
Parents who declined participation (*n* = 8)	“This would be too much for him to handle right now.” “The questions in this survey would upset him.”
Parents whose Adolescents and Young Adults (AYAs) declined participation (*n* = 22)	“I think this might help him, but he gets to decide.” “Is there a similar study for parents?”
AYAs who declined participation (*n* = 22)	“I don’t believe in this psych crap.” “I am not interested in doing that.”
Enrolled AYAs regarding choice of digital or paper-pencil survey	Baseline (*n* = 92): 23 (25%) prefer digital; 69 (75%) prefer paper-pencil 6-month (*n* = 74): 50 (68%) prefer digital; 24 (32%) prefer paper-pencil 12-month (*n* = 67): 58 (87%) prefer digital; 9 (13%) prefer paper-pencil

**Table 2:** Characteristics of participants who (**A**) enrolled and completed surveys, (**B**) enrolled and did not complete surveys, and (**C**) who declined enrollment.

	A. AYAs Who Enrolled and Completed Surveys (*n* = 92)			B. AYAs Who Enrolled and Did Not Complete Surveys (*n* = 7)			C. AYAs Who Were Approached and Declined Enrollment (*n* = 30)
Characteristic	Usual Care (*n* = 44)	PRISM (*n* = 48)	All [*n* = 92]	Usual Care [*n* = 6]	PRISM [*n* = 1]	All [*n* = 7]	All [*n* = 30]
	(*n*, %)	(*n*, %)	(*n*, %)	(*n*, %)	(*n*, %)	(*n*, %)	(*n*, %)
Female	24 (55)	16 (33)	40 (43)	2 (33)	0 (0)	2 (29)	6 (20)
12-17 years-old at enrollment	32 (73)	35 (73)	67 (73)	6 (100)	1 (100)	7 (100)	22 (73)
18-25 years-old at enrollment	12 (27)	13 (27)	25 (27)	0 (0)	0 (0)	0 (0)	8 (27)
Non-White Race	19 (43)	15 (31)	33 (36)	4 (67)	0 (0)	4 (57)	6 (20)
Leukemia/Lymphoma	27 (61)	30 (63)	57 (62)	4 (67)	1 (100)	5 (71)	17 (57)
Central Nervous System (CNS)	3 (7)	3 (7)	6 (7)	1 (17)	0 (0)	1 (14)	3 (10)
Non-CNS Solid Tumor	14 (32)	15 (31)	29 (32)	1 (17)	0 (0)	1 (14)	10 (33)
Advanced Cancer at Enrollment	14 (32)	10 (21)	24 (26)	3 (50)	1 (100)	3 (43)	11 (37)

PRISM: Promoting Resilience in Stress Management.

**Table 3:** Enrollment Rates by time since hearing diagnostic news.

	1–5 Weeks after Diagnostic News	6–10 Weeks after Diagnostic News
**New Diagnosis of Cancer**		
Number enrolled	53	20
Number approached	67	25
Enrollment Rate	79	80
**New Diagnosis of Recurrent, Progressive, or Refractory Cancer (“Advanced” Cancer)**		
Number enrolled	10	17
Number approached	18	20
Enrollment Rate	56	85
**Total**		
Number enrolled	63	37
Number approached	85	45
Enrollment Rate	74	82

**Table 4:** Observations by study stage and lessons learned for future research.

Study Stage	Observations *(Source)*	Lessons Learned for Future Research
**Screening and Approach**	AYAs could not be approached if their parents were not at the bedside. *(CRC field notes)*	Consider alternative approaches for parents/guardians who are unable to be at the bedside. These may include phone-based consent conferences and/or after-hours staffing.
	“Flexibility was key. We were most successful when we met families where they were at–both physically, like where they were in the hospital (clinic or infusion or the patient room) and also where they were emotionally. We learned how to read the room to know what to say and when.” *(CRC focus group)* “It took time and patience and presence. Time and patience to wait for patients to be available, and presence to listen and engage.” *(CRC focus group)*	Accrual was successful because there was a person in the hospital/clinic every day. This may require a large, flexible, research team or sharing staff resources with other research programs.
	“Relationships matter. Nurses, [Medical Assistants] and other staff helped me know when to approach them.” *(CRC focus group)* “If they had an ‘information heavy’ day, then I tried not to approach.” *(CRC focus group)*	Collaboration with clinical teams can help staff time approaches.
	“You always have to ask. You have to know your biases. What you think is a bad day to approach might not be a bad day for them.” *(CRC focus group)* “I had to learn how to navigate the medical chart.” *(CRC focus group)*	Non-clinical staff may benefit from additional training and support regarding how to interact with ill patients.
**Enrollment**	“Emphasizing the fact that we wanted to learn from the AYAs, that their voices mattered, was the most important factor [to explain our high enrollment rate].” *(CRC focus group)* “It helped that they saw the same person each time. It promoted trust.” *(CRC focus group)* “I said, ‘you know, we really haven’t figured this out yet, and we know we can do better,’ and then the AYA and the parent would start to nod.” *(CRC focus group)*	Engaging the AYA and family about how their voice and story is valuable, and validating their experience while building rapport, may encourage enrollment.
	Several parents wanted their AYA children to participate, even when the children declined. *(CRC field notes)* Parents and AYA patients declined participation for different reasons: parents who declined were commonly concerned about the burden of the intervention; AYAs declined because of disinterest or the stigma of mental health interventions. *(CRC field notes)* “Having a ‘script’ helped. Like, when they said, ‘I already know how to cope,’ I would say, ‘Great! You are exactly the person we want to learn from!’ And then they would start to listen.” *(CRC focus group)*	Joint discussions about the study, its research question and rationale, and its requirements may help patients and families make decisions together. When staff take time to explore individual and family concerns, more agree to participate.
	AYAs with new cancer were just as likely to enroll within the first few weeks of their diagnosis as later. AYAs with advanced cancer more commonly enrolled >4 weeks after their cancer had progressed. *(CRC field notes)*	Timing of approach may vary for different patient groups. Hearing of a recurrence may demand more processing time.
**Baseline Data Collection**	More study participants declined further participation when randomized to the inactive control arm. *(CRC field notes)* “They were disappointed when they didn’t get PRISM.” *(CRC focus group)*	Collect baseline data (including surveys) prior to randomization.
		Consider “active” control arms so participants in both arms “receive” something.
**Study Observation Period**	Death and critical illness were prevalent in this population of AYAs with cancer. *(CRC field notes)*	Study staff must be prepared for uncontrollable attrition and incomplete intervention delivery. Allowing flexibility in study procedures (e.g., allowing surveys to be collected late and/or intervention sessions to be rescheduled) may facilitate retention.
	Only 1 intervention recipient choose to discontinue participation once they started the intervention. *(CRC field notes)*	Consider strategies to engage participants early (e.g., trust and rapport building); once they start, they are more likely to finish.
**Outcome Data Collection**	Most participants who were still alive/well-completed surveys at 6-months. Their preferences for data collection (digital versus paper-pencil) evolved. “They started doing more digital because we started offering them an iPad to do the survey in real time. I think they just wanted to do whatever was most convenient.” *(CRC focus group)*	Consider patient and staff convenience. Digital survey completion may be facilitated by providing equipment to respond in real time.
**All Stages**	“Screening, coordinating approaches, going back and forth to discuss enrollment, getting to know families, delivering interventions, and monitoring survey collection all took a lot of time. It worked because we could be fully devoted to the project.” *(CRC focus group)*	Successful conduct of a clinical trial may require at least one CRC to be devoted to the project full-time.
	“It was much harder for me because I was not working full time with the team. When I started taking on more responsibility, when I became more invested in the impact of the work we do, I also got more enthusiastic.” *(CRC focus group)* “Connectedness and support within the research team and to the clinical teams helped. We were all working towards the same goal.” *(CRC focus group)*	Intra-team engagement boosts morale and study success. Consider embedded team-building activities and opportunities to encourage staff ownership of the project.
